# Nucleotide diversity of vernalization and flowering-time-related genes in a germplasm collection of meadow fescue (*Festuca pratensis* Huds. syn. *Lolium pratense* (Huds.) Darbysh.)

**DOI:** 10.1002/ece3.828

**Published:** 2013-10-09

**Authors:** Hiroshi Shinozuka, Melanie L Hand, Noel O I Cogan, German C Spangenberg, John W Forster

**Affiliations:** 1 Biosciences Research Division, Department of Environment and Primary Industries, AgriBio, The Centre for AgriBioscience5 Ring Road, La Trobe University Research and Development Park, Bundoora, Victoria, 3083, Australia; 2 Dairy Futures Cooperative Research CentreBundoora, Victoria, 3086, Australia; 3 La Trobe UniversityBundoora, Victoria, 3086, Australia

**Keywords:** Casein kinase II α-subunit, *CONSTANS* (*CO*), *FT1* (*Vrn3*), MADS-box (*Vrn1*), natural selection

## Abstract

In plant species, control of flowering time is an important factor for adaptation to local natural environments. The *Vrn1,* *CO,* *FT1* and *CK2α* genes are key components in the flowering-specific signaling pathway of grass species. Meadow fescue is an agronomically important forage grass species, which is naturally distributed across Europe and Western Asia. In this study, meadow fescue flowering-time-related genes were resequenced to assess nucleotide diversity in European and Western Asian subpopulations. Identified sequence polymorphisms were then converted into PCR-based molecular genetic markers, and a meadow fescue germplasm collection was genotyped to investigate global allelic variation. Lower nucleotide diversities were observed for the *Vrn1* and *CO* orthologs, while relatively higher values were observed for the *FT1* and casein kinase II α-subunit (*CK2α*) orthologs. The nucleotide diversity for *FT1* orthologs in the Western Asian subpopulation was significantly higher than those of the European subpopulation. Similarly, significant differences in nucleotide diversity for the remaining genes were observed between several combinations of subpopulation. The global allele distribution pattern was consistent with observed level of nucleotide diversity. These results suggested that the degree of purifying selection acting on the genes differs according to geographical location. As previously shown for model plant species, functional specificities of flowering-time-related genes may also vary according to environmental conditions.

## Introduction

Control of flowering time is essential for adaptation of plant species to natural environments (Shimizu et al. 2011). Variation for flowering time is largely governed by genetic factors, and genes involved in this trait have been identified from model plant species through a combination of molecular biological and genetic approaches, such as mutagenesis, transgenic modification and gene expression profiling (Andres and Coupland 2012). In *Arabidopsis thaliana* (L.), the *CONSTANS* (*CO*), *FLOWERING LOCUS T* (*FT*) and *FLOWERING LOCUS* C (*FLC*) genes are important components of the flowering-specific signaling pathway. Expression of the *CO* gene, which encodes a B-box zinc-finger protein containing a CONSTANS, CONSTANS-LIKE and TIMING OF CAB EXPRESSION1 (CCT) domain, is enhanced under long-day conditions, leading to accumulation of the cognate protein. The CO protein is believed to directly bind to the promoter of the *FT* gene to activate expression. The FT protein, which shows similarity to phosphatidylethanolamine-binding proteins, is essential for floral transition at the shoot apical meristem. In contrast to *CO*, the FLC protein, which is a MADS-box transcription factor, inhibits expression of the *FT* gene through direct binding to the relevant promoter.

In rice (*Oryza sativa* L.), which serves as a model grass (Poaceae) plant species, genes involved in the flowering-specific signaling pathway were identified through map-based cloning of heading date-related quantitative trait loci (QTLs; Tsuji et al. 2011). The genes corresponding to the *Hd1*, *Hd3a* and *Hd6* loci were found to encode CO-like, FT-like and casein kinase II α-subunit (CK2α) proteins, respectively. The CK2α protein enhances function of the CO-like protein through indirect interaction under short-day, but not long-day conditions. Although the CO-like protein regulates expression of the *FT*-like gene, similar to the activity of the *A. thaliana* protein, function varies according to environmental conditions, leading to promotion of *FT*-like gene expression under short-day conditions, and repression under long-day conditions. As well as allelic variation in natural populations, the functional flexibility of flowering-time genes is thought to have contributed to adaptation to local environments (Izawa 2007).

Although the molecular components of the flowering-specific signaling pathway are also widely conserved in nonmodel plant species, the functional specificities of those genes occasionally differ (Trevaskis et al. 2007; Andres and Coupland 2012). Cereal and forage species in the Pooideae (cool-season) subfamily of the Poaceae require vernalization in order to induce a transition from the vegetative to reproductive developmental phase. In wheat (*Triticum aestivum* L.) and barley (*Hordeum vulgare* L.), which are representative members of the Pooideae tribe Triticeae, vernalization requirement is largely controlled by three loci, *Vrn1*, *Vrn2* and *Vrn3*, of which *Vrn1* and *Vrn3* encode MADS box and FT-like proteins, respectively. Expression of the *FT*-like gene (*FT1*) is repressed by the *Vrn2* protein during spring and autumn (long-day condition), and expression of *Vrn1* is induced in winter, leading to repression of the *Vrn2* gene. Due to the function of the Vrn1 protein, *FT1* gene expression is induced to promote development of reproduction organs in spring (Trevaskis et al. 2007).

Among turf and forage grass species of the Pooideae tribe Poeae, flowering-time-related traits have been extensively studied in perennial ryegrass (*Lolium perenne* L.). Orthologs for the *Vrn1*, *CO*, *FT1 and CK2α* genes have been cloned and characterized from this species (Jensen et al. 2005; Shinozuka et al. 2005; Armstead et al. 2008; Studer et al. 2012). Colocation on genetic linkage maps between the gene ortholoci and flowering time QTLs has been demonstrated, suggesting conservation of key genes in the flowering-specific pathway. DNA sequence polymorphisms in the perennial ryegrass *Vrn1* and *FT1* gene orthologs were determined in order to identify “diagnostic” polymorphisms, diversity for which was demonstrated to be significantly correlated with flowering time variation (Asp et al. 2011; Skøt et al. 2011). Due to a significant correlation between flowering time-related characters and vegetative biomass-related traits, knowledge of the flowering time-related gene alleles may be exploited in order to improve the biomass yield of turf and forage grass cultivars (Yamada et al. 2004).

Meadow fescue (*Festuca pratensis* Huds. syn. *Lolium pratense* (Huds.) Darbysh.) is an outbreeding diploid (2*n* = 2*x* = 14) member of the Poeae tribe, which is widely sown as a pasture crop in northern temperate regions (Ergon et al. 2006). This species is closely related to perennial ryegrass (Hand et al. 2010, 2012a,b), such that construction of a genetic linkage map containing functionally associated markers demonstrated genome-wide collinearity between the two taxa (Alm et al. 2003). Genetic factors controlling agronomically important traits, such as drought and frost tolerance, flowering time and seed production-related characters, have been investigated through the use of biparental mapping populations (Ergon et al. 2006; Alm et al. 2011). A QTL identification study suggested the presence of multiple loci related to vernalization requirement on chromosome 4F, to which the *Vrn1* ortholog was assigned (Ergon et al. 2006).

Both meadow fescue and perennial ryegrass are widely sown in Europe and Western Asia, and the geographical distribution structures of organelle genome-specific haplotypes of the two species have been investigated (Balfourier et al. 2000; Fjellheim et al. 2006). While no clear association between the distribution structure of meadow fescue chloroplast DNA haplotypes and human migration routes was observed, such a relationship was inferred for perennial ryegrass (Balfourier et al. 2000). In contrast, the previous phylogeographic studies suggested that the distribution structure of meadow fescue genotypes shows a weaker relationship with agricultural practices in Europe and Western Asia and that this species is more naturally distributed than perennial ryegrass (Fjellheim et al. 2006). Recently, worldwide germplasm collections of both meadow fescue and tall fescue (*Festuca arundinacea* Schreb. syn. *Lolium arundinaceum*) curated by the United State Department of Agriculture-Agricultual Research Service (USDA-ARS) were genotyped through resequencing of the chloroplast genome-located *matK* gene and the nuclear ribosomal DNA internal transcribed spacer (nrDNA ITS), permitting precise species-specific identification within the complex germplasm collections (Hand et al. 2012b). This study identified 189 distinct meadow fescue genotypes originating from 27 countries within the two collections (Hand 2012c), providing a geographically diverse core resource for studies of relationships between genetic and ecoclimatic variation.

In this study, nucleotide diversity of meadow fescue flowering-time-related genes has been assessed. Regions of the meadow fescue *Vrn1*, *CO*, *FT1 and CK2α* orthologs (*Fp*Vrn1, *Fp*CO, *Fp*FT1 and *Fp*CK2α) were resequenced, and nucleotide diversity within three sample groups was evaluated. Population-specific single nucleotide polymorphisms (SNPs) were subsequently converted into cleaved amplified polymorphic sequence (CAPS) markers, and the meadow fescue germplasm collection was extensively genotyped in order to investigate global allele distribution. As flowering-time-related genes are key components of natural environment adaptation, it was hypothesized that such genes have been subjected to natural selection pressures due to ecoclimatic factors and that the degree of selection strength may vary according to geographical location. The results of this analysis are presented and discussed.

## Materials and Methods

### Plant materials and DNA extraction

Meadow fescue seed was obtained from the USDA-ARS collection (Hand et al. 2012b). Single seeds from each accession were germinated and grown under controlled conditions. Meadow fescue genotypes were selected and classified into six groups on the basis of geographical origin (Hand et al. 2012b): Northern Europe (NE), Russia and former Soviet Union (R), Central Europe (CE), Southern Europe (SE), Western Asia (WA) and the rest of the world (RW). A subset of 36 genotypes was then selected to represent each group, with an emphasis on origin from Western Eurasia (Table 1). The NE group and a combination of the Central and Southern European groups (CSE) each contributed 11 individuals. Members of the NE group were collected from three countries located between latitudes 55 and 65°N, while the CSE combined group contained members of accessions from four countries located between latitudes 40 and 50°N. The WA group consisted of 10 individuals that originated from 4 countries located between latitudes N 30 and 40°N. A single individual from Russia (56.9°N) represented the R group, while a single genotype from Japan (43.1°N) and two individuals from Australia represented the RW group. DNA was extracted from a single young leaf of each plant using the DNeasy 96 Plant Kit (QIAGEN, Hilden, Germany). Simple sequence repeat (SSR) genotyping data from the previous study were used for assessment of genetic diversity of the selected genotypes (Hand et al. 2012b). The DARwin5 program was used for the construction of a genetic dissimilarity matrix and radial unweighted neighbor-joining tree (Perrier et al. 2003).

**Table 1:** Meadow fescue accessions used for resequencing analysis

		Origin country			
		
Group	ID	Country	Locality	Latitude	Longitude
Northern Europe (NE)	372623	Denmark	n.d.	56.2	9.4
	372624	Denmark	n.d.	56.2	9.5
	577107	Norway	n.d.	61.5	10.2
	595035	Norway	n.d.	58.3	6.7
	595036	Norway	n.d.	59.8	5.2
	595037	Norway	n.d.	61.1	8.6
	595038	Norway	n.d.	61.9	9.2
	595039	Norway	n.d.	61.3	9.9
	283305	United Kingdom	n.d.	n.d.	n.d.
	311050	United Kingdom	n.d.	n.d.	n.d.
	595019	United Kingdom	n.d.	n.d.	n.d.
Central and Southern European (CSE)	289005	Hungary	n.d.	n.d.	n.d.
	289013	Hungary	n.d.	n.d.	n.d.
	595025	Italy	n.d.	45.9	9.9
	595028	Romania	n.d.	47.8	26.1
	595029	Romania	n.d.	47.6	26.3
	595031	Romania	n.d.	47.9	23.9
	595032	Romania	n.d.	47.9	23.9
	595033	Romania	n.d.	45.9	25.8
	234881	Switzerland	Wildhaus	47.2	9.3
	577102	Switzerland	n.d.	46.2	6.9
	595018	Switzerland	n.d.	46.6	6.9
Western Asia (WA)	221927	Afghanistan	Bamian, Kabul	34.5	69.2
	275335	Afghanistan	Ghanzi	n.d.	n.d.
	317425	Afghanistan	Qala Sakawa	35.2	67.5
	229500	Iran	Dastana, south of Shahr-Kord	32.3	50.9
	229692	Iran	41 miles north of Kermanshah along road to Sanandaj	35.1	47.0
	380858	Iran	Divandarrh	35.9	47.0
	384859	Iran	70 km north of Semnan	36.3	53.4
	204447	Turkey	Near Pinarbasi, Kayseri.	38.7	36.4
	383647	Turkey	Erzincan, 28 km toward Baskoy	39.7	39.5
	502378	Uzbekistan	Samarkand	39.7	67.0
Russia and former Soviet Union (R)	315445	Former Soviet Union	Krasnodar Territory	56.9	91.9
Rest of the world (RW)	438525	Japan	Sapporo	43.1	141.4
	283310	Australia	n.d.	n.d.	n.d.
	283316	Australia	n.d.	n.d.	n.d.

Genotypes from Russia, Japan and Australia were assembled into the NE, CSE and WA groups, respectively, according to latitude of origin. n.d., Data not available.

### Primer design and PCR amplification

PCR primers were designed using the Sequencher™ software version 4.7 for Windows (Genecodes, Ann Arbor, MI) and Oligo Calc program (Kibbe 2007). Primers for the *Fp*CO and *Fp*FT1 genes were designed on the basis of the previously sequenced meadow fescue *CO* and *FT1* orthologs, respectively (NCBI GenBank ID: AJ833018 and FN993915; Armstead et al. 2005; Skøt et al. 2011). As sequence information for the *Fp*Vrn1 and *Fp*CK2α genes was not directly available, DNA sequences from related species were obtained (JN969602, JN969603, FJ793194, AB213316, AK354232 and XM_003559017) and aligned using the Sequencher™ software. Primers capable of cross-specific amplification were then designed within the conserved regions. PCR amplification was performed using Immolase™ DNA polymerase (BIOLINE, London, UK), following manufacturer's instructions. The PCR amplicons were visualized on a 2.0% (w/v) agarose gel containing 0.5× SYBR® Safe DNA gel staining (Life Technologies, Carlsbad, CA).

### Sanger PCR-direct sequencing

The PCR products were treated with exonuclease I (0.5 U) and shrimp alkaline phosphatase (0.5 U) at 37°C for 40 min, and the enzymes were then heat deactivated at 85°C for 20 min. Direct Sanger sequencing analysis was performed using BigDye™ terminator chemistry (Life Technologies), following the manufacturer's instructions, and the fluorochrome-labelled fragments were size-separated on the ABI 3730xl Prism sequencer (Life Technologies).

### DNA sequence data analysis

Sequence reads were aligned and manually corrected using Sequencher (Genecodes), and nucleotide polymorphisms were identified. An unphased FASTA file of the Sanger sequence reads was prepared and imported into the DnaSP v5 program (Librado and Rozas 2009). Haplotype reconstruction was performed with the PHASE algorithm (Stephens et al. 2001; Stephens and Donnelly 2003), and nucleotide diversity (π) was subsequently calculated. Significantly different combinations of nucleotide diversity were identified with the Tukey–Kramer method. A studentized range (*q*) value of 4.46 was used for the Tukey–Kramer method at the significance level of α = 0.01.

### CAPS assay

PCR amplicons that included polymorphic nucleotide sites were generated with locus-specific primers. The PCR products (5 μL) were digested with restriction enzyme (0.5 U), following the manufacturer's instruction. The DNA fragments were visualized on a 2.0% (w/v) agarose gel with 0.5× SYBR® Safe DNA gel staining.

## Results

### SSR-based genetic diversity within the sample set

**Figure 1:**
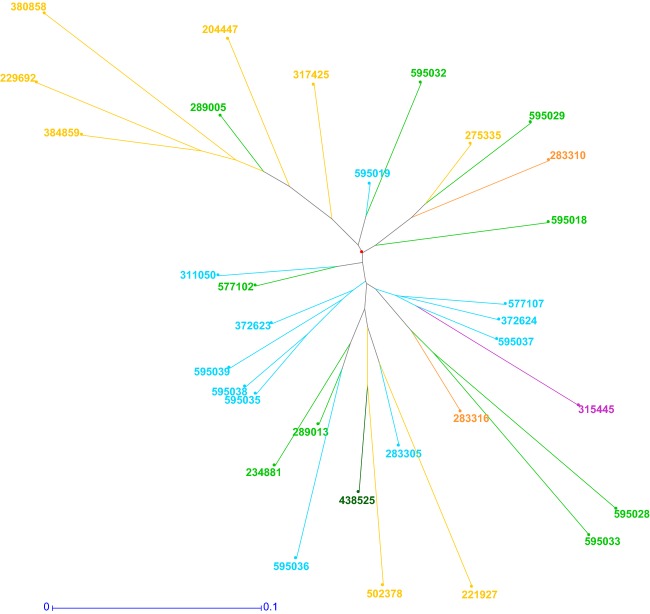
Unweighted neighbor-joining tree constructed from genotypic analysis of 32 meadow fescue genotypes based on data from 20 simple sequence repeat markers. The individuals included in the Northern Europe, Central and Southern European and Western Asia groups are shown in light blue, green and yellow, respectively. The individuals from Russia, Japan and Australia are represented in purple, dark green and orange, respectively.

A neighbor-joining (NJ) tree was constructed for the 36 individuals that represent broad geographical origins, based on genotyping results from use of 20 genomic DNA-derived SSR markers (Fig. 1). The NJ analysis excluded two individuals from each of the CSE (595025 and 595031) and WA (229500 and 383647) groups from the dendrogram, but all individuals from Russia, Japan and Australia were included.

### PCR amplification and sequencing of flowering time-related genes

**Figure 2:**
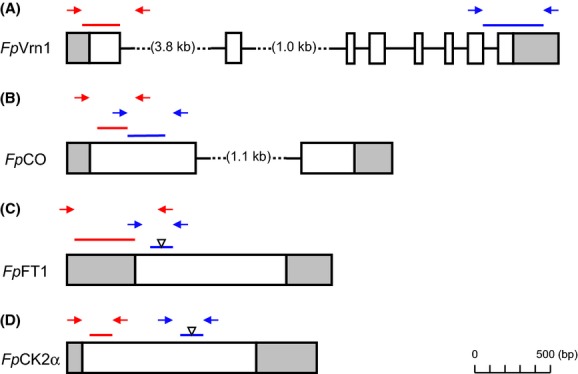
Graphical presentation of PCR primers and sequenced regions. The white and black boxes represent exon and 3′-untranslated regions, respectively. The black line between exons represents intron sequence, and the gap in intron is shown with the broken line. The red and blue arrows indicate PCR primers for regions 1 and 2, respectively, and the regions 1 and 2, which were used for the nucleotide diversity study, are shown with the red and blue lines. (A) PCR primers for the *Fp*Vrn1 gene based on genomic structure of the perennial ryegrass *Vrn1* ortholog. (B) The PCR primers for the *Fp*CO gene based on genomic structure of the meadow fescue *CO* ortholog. (C and D) The PCR primers for the *Fp*FT1 and *Fp*CK2α genes based on mRNA sequences of the perennial ryegrass *FT1* and *CK2α* orthologs, respectively. The white triangle shows the site of interpopulation of introns in sequenced regions.

The *Fp*Vrn1, *Fp*CO, *Fp*FT1 and *Fp*CK2α gene-related sequences were amplified using PCR with a total of eight primer pairs (two pairs of primers for each of the 4 genes; Table 2; Fig. 2). Amplicon sizes varied from *c*. 200 to 600 bp depending on primer combinations. The amplicons were sequenced, and their identity was confirmed on the basis of high sequence similarity to the previously sequenced *Vrn1*, *CO*, *FT1* and *CK2α* genes from meadow fescue and related species (Table 2). Putative exons, introns, 3′-untranslated regions (UTRs) and 5′-UTRs were included in the sequenced amplicons.

**Table 2:** PCR primer sequences for amplification from the flowering-time-related genes and similarity to the corresponding DNA sequence

Locus	Region	Primer	Sequence (5′ → 3′)	*E* value
*Fp*Vrn1	Region 1	f	TCTCCTCTTCTTCCCCACTG	2E−125 (FJ793194.1)
		r	GTCGGTTGCGAACTCGTAGA	
	Region 2	f	GAAGCTACCCCAGCAACAAA	4E−155 (FJ793194.1)
		r	GAGGGTCGTAGAAAGTGGAA	
*Fp*CO	Region 1	f	AGGCAGAAGAACAGAAGAAG	1E−117 (AJ833018.1)
		r	CACAGGCACGCACTGGTGCA	
	Region 2	f	GGTGCCAAGCGTGGTGTACT	3E−180 (AJ833018.1)
		r	GTAGGAATTGTACCCGACAA	
*Fp*FT1	Region 1	f	GCATCCCGTCCACATGATAG	0 (FN993923.1)
		r	ACGAGTGTGTAGAAGGTCCT	
	Region 2	f	GTTGGTGGTTGGTAGGGTTG	0 (FN993915.1)
		r	ACCAATGGAGGTATTCTCTG	
*Fp*CK2α	Region 1	f	CCGGAAACCCTAGCCCGC	5E−50 (AB213316.1)
		r	CTCGTAGTCCCAGTACTCCT	
	Region 2	f	GGCCCCAGTCTATCAAGCGA	1E−31 (AB213316.1)
		r	GTGCTCTATCCCACGTTGAC	

### DNA polymorphism identification and nucleotide diversity assessment

The Sanger sequence reads were aligned for SNP identification. Total of 21 and 18 SNP sites were identified in the *Fp*FT1- and *Fp*CK2α-related sequences, respectively (Table 3). All SNPs found in regions 1 and 2 of *Fp*FT1 and region 2 of *Fp*CK2α were located in either 5′-UTRs or introns. In the *Fp*CK2α-region 1, 10 exonic SNPs were identified, of which 7 were putatively nonsynonymous. Relatively few polymorphic sites were identified in the *Fp*Vrn1- and *Fp*CO-related sequences (Table 3). A single nonsynonymous SNP was found in the *Fp*Vrn1-related sequences, and the other SNPs were located in either 5′-UTR or intron. One synonymous SNP and three nonsynonymous SNPs were identified in the *Fp*CO-related sequences. No SNP-containing sites were identified in either *Fp*CO-related sequences of the NE group or region 1 of *Fp*Vrn1-related sequences from the CSE combined group. The highest SNP prevalence (10 across 147 bp) was found in region 1 of the *Fp*CK2α-related sequence. Additional SNPs were identified when sequencing data from the Russia-, Japan- and Australia-derived individuals were combined, corresponding to one and two SNPs in the *Fp*FT1- and *Fp*CO-related sequences, respectively (Table 3). No additional SNPs were, however, identified in the *Fp*Vrn1- and *Fp*CK2α-related sequences.

**Table 3:** Numbers of SNP and haplotype and nucleotide diversity (π) identified in the flowering-time-related genes

Locus	Region	Group	*n*	Length (bp)	SNP	Haplotype	π±SD	π with outer region-originated germplasm(s) (*n* = 12)
*Fp*Vrn1	1	NE	11	247	1	2	0.00037 ± 0.00033	0.00034
		CSE	11	247	0	1	0	0
		WA	10	247	1	2	0.00109 ± 0.00046*	0.00092
	2	NE	11	449	2 (1)	3	0.00147 ± 0.00024**	0.00139
		CSE	11	449	2 (1)	3	0.00102 ± 0.00027	0.00106
		WA	10	449	1 (1)	2	0.00060 ± 0.00025**	0.00051
*Fp*CO	1	NE	11	233	0	1	0**	0
		CSE	11	233	1 (1)	2	0.00039 ± 0.00035	0.00036
		WA	10	233	1 (1)	2	0.00081 ± 0.00046**	0.00137
	2	NE	11	264	0	1	0	0
		CSE	11	264	1	2	0.00034 ± 0.00031	0.00032
		WA	10	264	3	3	0.00181 ± 0.00108*	0.002
*Fp*FT1	1	NE	11	390	2	3	0.00159 ± 0.00050	0.00187
		CSE	11	386	3	4	0.00132 ± 0.00039	0.0014
		WA	10	386	7	7	0.00379 ± 0.00093*	0.00343
	2	NE	11	474	3	4	0.00196 ± 0.00042	0.00206
		CSE	11	474	4	4	0.00077 ± 0.00040	0.00071
		WA	10	474	14	6	0.00569 ± 0.00149*	0.00508
*Fp*CK2a	1	NE	11	147	8 (5)	3	0.02288 ± 0.00477	0.02524
		CSE	11	147	8 (5)	2	0.02827 ± 0.00206	0.02839
		WA	10	147	10 (7)	5	0.02875 ± 0.00370	0.02992
	2	NE	11	565	2	3	0.00071 ± 0.00023	0.00076
		CSE	11	565	1	2	0.00055 ± 0.00019	0.00051
		WA	9	565	8	4	0.00251 ± 0.00119*	0.00232

The number of nonsynonymous SNP is shown in brackets, following the total number of SNP. SNP, single nucleotide polymorphism; NE, Northern Europe; CSE, Central and Southern European; WA, Western Asia.

* Nucleotide diversity significantly different (α = 0.01) from the other two in the same category.

** Significantly different combination of nucleotide diversities in the same category (α = 0.01).

Nucleotide diversity was calculated for each gene sequence and each geographical subpopulation. Diversity values were then compared between subpopulations to investigate the association between flowering-time diversity and geographical origin. The highest level of nucleotide diversity was observed in the *Fp*CK2α-region 1 sequence, more than 10 times higher than that of the remainder of this gene (Table 3). However, no significant differences between groups were found in the sequence of this region. The nucleotide diversity in five sequenced regions for the WA group was significantly higher than those of the NE and CSE groups. The exception was in *Fp*Vrn1-region 2, for which the NE group significantly exceeded the WA group. Levels of nucleotide diversity were largely unchanged when data from genotypes originating from more distant localities (Australia, Japan and Russia) were included.

### Allele variation in flowering-specific genes

**Figure 3:**
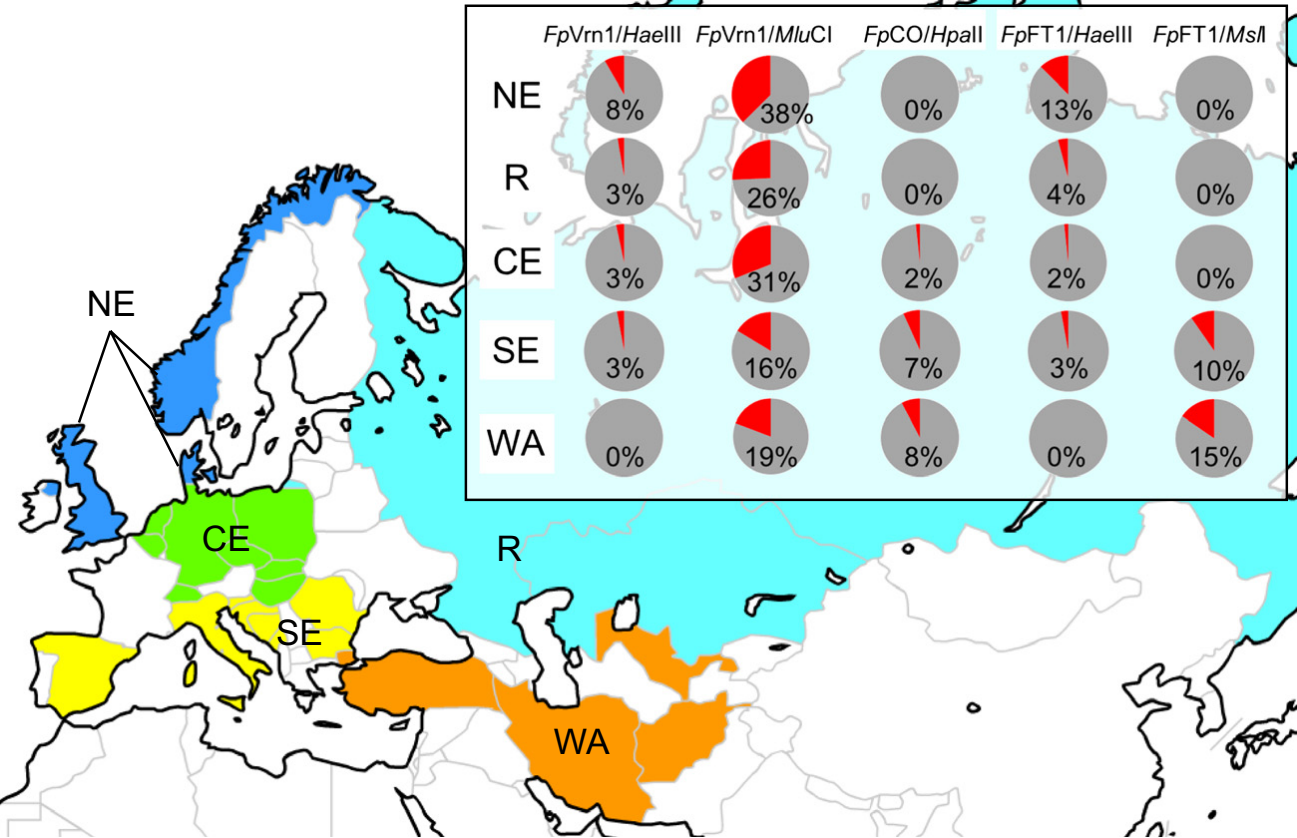
Geographical distribution of regions contributing to each group, and graphical representation of cleaved amplified polymorphic sequence (CAPS) allele frequency within each group. Countries classified into the Northern Europe (NE), Russia and former Soviet Union (R), Central Europe (CE), Southern Europe (SE) and Western Asia (WA) groups are shown in dark blue, green, light blue, yellow and orange, respectively. The CAPS allele frequencies are shown in pie-chart format, gray and red colors representing frequencies of the major and minor alleles, respectively. The number in each pie-chart shows the frequency of the minor allele.

In order to screen a larger number of accessions in a high-throughput fashion, sequence polymorphisms were converted into CAPS assays. A total of five polymorphic sites capable of conversion into CAPS markers were identified across *Fp*Vrn1-region 2, *Fp*CO-region 2 and *Fp*FT1-region 2 (Table 4), and the resulting genotypic assays were performed across all 152 meadow fescue individuals (Fig. 3; Tables S1 and S2). The minor (lower frequency) alleles of the *Fp*Vrn1/*Hae*III and *Fp*FT1/*Hae*III markers, which were identified only in the NE group during resequencing, were found in the Russian and each of the European groups. The minor alleles of the *Fp*CO/*Hpa*II and *Fp*FT1/*Msl*I markers were found in the CSE and WA groups used in the reseqeuncing assay. Although one individual with the minor *Fp*CO/*Hpa*II allele was found in the CE group, no genotypes from the NE and R groups contained these alleles. The minor *Fp*FT1/*Msl*I allele was present in additional individuals of the SE and WA groups, but not the R and other European groups. The minor allele of the *Fp*Vrn1/*Mlu*CI marker was found in all groups, and the frequencies were relatively high in the NE and CE as compared to SE and WA groups.

**Table 4:** Primer and restriction enzyme combination for cleaved amplified polymorphic sequence (CAPS) analysis and allele frequency in the Northern Europe (NE), Central and Southern European (CSE) and Western Asia (WA) groups

	PCR primer			Alleles		Frequency of minor allele (%)			
				
CAPS marker	Forward (5′ → 3′)	Reverse (5′ → 3′)	Restriction enzyme (recognition site)	Major	Minor	NE (*n* = 12)	CSE (*n* = 12)	WA (*n* = 12)	All (*n* = 36)
*Fp*Vrn1/*Hae*III	AGCTCTTCCTCCTCCTCCTT	GCATCCTCTGCTCTGTCGCT	*Hae*III (GGCC)	Digested	Undigested	8.3	0	0	2.8
*Fp*Vrn1/*Mlu*CI	AGCTCTTCCTCCTCCTCCTT	GCATCCTCTGCTCTGTCGCT	*Mlu*CI (AATT)	Undigested	Digested	33.3	20.8	12.5	22.2
*Fp*CO/*Hpa*II	CTGTGGTTTCGCACACGGCCACAGCCATT	GTAGGAATTGTACCCGACAA	*Hpa*II (CCGG)	Undigested	Digested	0	4.2	12.5	5.6
*Fp*FT1/*Hae*III	AGGACCTTCTACACACTCGT	CCATCACCTGCGCATGTACA	*Hae*III (GGCC)	Digested	Undigested	16.7	0	0	5.6
*Fp*FT1/*Msl*I	AGGACCTTCTACACACTCGT	CCATCACCTGCGCATGTACA	*MsI*I (CAYNNNNRTG)	Digested	Undigested	0	4.2	8.3	4.2

## Discussion

### Linkage disequilibrium in meadow fescue

Natural selection exerts effects not only on mutations that contribute to trait-specific variation, but also noncausal nucleotide changes that are physically linked to the causal mutation site (Futuyma 1998; Frankham 2012). The extent of the sequence region affected by this “indirect” selection is determined by the decay of linkage disequilibrium (LD), and adjacent nucleotides within an area of high LD are expected to be subjected to a similar selective pressure. For grasses of the Poeae tribe, extent of LD has been directly studied in perennial ryegrass. Due to an obligate outbreeding reproduction habit, LD in perennial ryegrass exhibits rapid decay, typically over distances 500–2000 bp in length, comparable to the size of a single gene locus (Ponting et al. 2007; Fiil et al. 2011). In the absence of direct empirical data, it seems likely that LD decay would occur over equivalent physical distances in meadow fescue, due to similarities of outbreeding reproductive habit and genome size (Smarda et al. 2008). For this reason, identification and characterization of gene-specific sequence polymorphisms provide a possible means for tracing causal variation in response to ecoclimatic factors for species such as meadow fescue.

In the present study, nucleotide diversities between the two regions of each of the *Fp*Vrn1, *Fp*CO and *Fp*FT1 genes were not substantially different, consistent with a common selection history. In contrast, a large difference in nucleotide diversity was demonstrated between the two regions of the *Fp*CK2α-1 gene, suggesting that different selective pressures may have arisen between the regions. A more extensive resequencing activity over a larger sample of loci may be required to determine the average extent of LD within meadow fescue genes, and whether the flowering-time-related genes in the present study provide a representative group.

### Response of flowering time genes to ecoclimatic variation

Recent studies have demonstrated that the gene products involved in the flowering-specific signaling pathway may alter in terms of functionality depending on environmental conditions, especially latitudinal variations of seasonal day length (Tsuji et al. 2011). Previous trait-dissection studies in perennial ryegrass suggested that functionality of the *Vrn1* ortholog is predominant at middle latitudes (35.5°N), while that of the *FT1* (*Hd3a*) ortholog is more important at high latitudes (52.4°N; Yamada et al. 2004; Armstead et al. 2008; Shinozuka et al. 2012). In the present study, nucleotide diversity of meadow fescue flowering-time-related genes was determined and interpreted, based on the following hypotheses. Firstly, flowering-time-related genes are key determinants of adaptation to natural environments, through influence on the probability and timing of reproduction (Shimizu et al. 2011) and secondly, the functionality of meadow fescue flowering-time-related genes differs depending on latitudinal variation. Based on these assumptions, flowering-time-related genes may be subjected to selective sweeps leading to homogenization of genetic variants, due to a requirement for synchronization of flowering time, and differences of the degree of selective pressure may hence be detected as variations of nucleotide diversity.

In this study, nucleotide diversity among Europe- and West Asia-derived genotypes was investigated. As meadow fescue exhibits a relatively natural distribution within these areas (Fjellheim et al. 2006), correlations between gene-specific diversity and ecological parameters may be anticipated for the relevant genotypes. Understanding of such correlations may support the identification of favorable alleles at target genes for use in breeding activities. For example, desirable flowering-time-related gene alleles may be effectively sourced from populations with high levels of nucleotide diversity, and such alleles can be used to increase yield of vegetative biomass.

### SSR-based genetic diversity within meadow fescue germplasm

Genomic DNA-derived SSR genotyping data revealed the extent of genetic distance between the germplasm sources used for the resequencing activity The majority of these markers are derived from noncoding regions, which are expected to affected to a lesser extent than genic regions by selective pressures, hence permitting a less-biased assessment of affinity. Although some individuals from nearby locations were clustered together, individuals from each group were widely distributed across the phenetic tree. These data suggest that the candidates for genotypic analysis would be relatively unbiased in terms of background genetic structure, permitting assessment of gene-specific divergence.

### Nucleotide diversity of flowering-time-related genes

Limited nucleotide diversity in the *Fp*Vrn1 and *Fp*CO gene-related sequences was identified across the three groups, suggesting that strong selective pressures may have been exerted on these loci. Nonetheless, some significant differences across geographical combinations were identified. As the number of SNPs identified was small, further analysis is essential in order to establish a convincing correlation with geographical provenance. In contrast, relatively high SNP numbers were found in the *Fp*FT1 and *Fp*CK2α-1 gene-related regions. The significant difference in nucleotide diversity observed in *Fp*FT1-related sequences suggested that different selective pressures may have arisen between lower (30–40°N) and higher (40–65°N) latitudes. As QTL detection studies in perennial ryegrass have shown, the importance of the *FT1* ortholog may be greater in higher latitude regions. Similarly, in *Fp*CK2α-1 gene region 2, the nucleotide diversity of the WA group was significantly higher than those of the other groups. The nucleotide diversity in the region 1 of the same gene was not, however, significantly different at the level α = 0.01, possibly due to rapid decay of LD across this gene unit. A previous study (Fiil et al. 2011) described a higher degree of nucleotide diversity in perennial ryegrass flowering-time-related genes, including the *Vrn1* (π = 0.01992) and *CO* (π = 0.00119) orthologs, than in the present study. This difference may, however, be due to the influence of differing population structures, and different target regions of the orthologous genes selected for resequencing.

Due to the outbreeding reproductive habit, background genetic diversity of meadow fescue is relatively high within populations and hence relatively low between populations (Fjellheim and Rognli 2005). The genomic DNA-derived SSR genotyping data revealed a large level of genetic diversity within populations, supporting this scenario. Conversely, the nucleotide diversities of flowering-time-related genes were very limited, and significant differences were observed between populations. Such a difference may be due to distinct mechanisms of allelic diversification between gene-associated and noncoding DNA sequences. Further studies are required to explore this issue.

### Global allele distribution of flowering-time genes

The CAPS marker assay permitted simple and cost-effective genotyping for the larger sample set. Although a relatively small number of samples were used in the resequencing analysis, the allele frequencies were largely similar to those obtained from the expanded set, suggesting that alleles at low frequency (*c*. 5%) may be effectively identified from a relatively small number of genotypes. In the resequencing assay, the minor alleles at the *Fp*Vrn1/*Hae*III, *Fp*CO/*Hpa*II, *Fp*FT1/*Hae*III and *Fp*FT1/*Msl*I sites exhibited group specificity, and a similar distribution pattern was also observed for the CAPS genotyping assay (Table 4; Tables S1 and S2; Fig. 3). The relative frequencies by group of the minor alleles for *Fp*Vrn1- and *Fp*CO-specific CAPS markers were consistent with observed levels of nucleotide diversity. A relatively high frequency of the *Fp*FT1/*Msl*I minor allele was found in the Western Asia-derived genotypes, for which the highest nucleotide diversity of the *Fp*FT1 gene was observed. The frequency of the *Fp*FT1/*Hae*III minor allele was, however, higher in the NE and R groups. The distribution pattern of the minor alleles may hence represent different and distinct evolutionary histories of the *Fp*FT1 gene between the Europe- and Western Asia-derived individuals. All minor alleles were identified in the SE group. In particular, higher frequencies of the minor alleles were found in genotypes from the former Yugoslavia (*n* = 22) and in Italy (*n* = 5; Table S2), suggesting that genetic diversity of the flowering-time-related genes may be enhanced within these localities.

The RW group consisted of genotypes derived from Australian, Canadian and Japanese origins. This study suggested that the genetic background of these genotypes was not substantially different from that of Europe and West Asia-origin genotypes, despite large geographical separation. This is probably because meadow fescue has been introduced into these nations from Europe within the relatively recent past (Casler and van Santen 2001; Hand 2012c).

### Conclusions and future directions

In this report, the first study of nucleotide diversity within meadow fescue flowering-time-related genes has been provided. Through resequencing and CAPS genotyping activities, the following insights were obtained as follows: (1) nucleotide diversity in the flowering-time-related genes may be limited, especially in the *Fp*Vrn1 and *Fp*CO genes; (2) nucleotide diversity in the genes may be more extensive in South European populations; (3) LD in meadow fescue may decay rapidly, similar to the situation in other outbreeding plant species; and (4) the degree of nucleotide diversity in the flowering-time-related genes may differ depending on latitudinal position.

Although some data in support of the original hypotheses were obtained, several problems remain. Firstly, the nature of the environmental variation that affects nucleotide diversity is unclear. In outbreeding plant species, synchronization of flowering time is essential for mating success (Shimizu et al. 2011). In the previously described perennial ryegrass-based trait-dissection study, the maximum LOD scores for both seed set and heading date QTLs were observed to co-locate with the *FT1* ortholog-derived marker (Armstead et al. 2008), suggesting a close relationship between the two processes. However, flowering-time-related genes have also been suggested to contribute to low temperature stress tolerance, as vernalization requirement genes are believed to maintain high expression of cold tolerance-related genes (Prasil et al. 2004). In meadow fescue, such an association between the diversity of *Fp*Vrn1 gene and variation for frost tolerance has been reported (Alm et al. 2011). It is therefore possible that low temperature conditions, which also vary with latitude, rather than differences of seasonal day length were responsible for the nucleotide diversity differences observed in this study.

Secondly, although the relationship between flowering time and mating success has been considered at the intrapopulation level (Armstead et al. 2008; Shimizu et al. 2011), the relationship at the interpopulation level is still unclear. The meadow fescue genotypes analyzed in this study were collected from widespread areas, and so the data obtained in the current study represent estimates of nucleotide between, rather than within, natural populations. The effects of flowering time on mating success at the interpopulation level require further analysis, as well as the evolutionary histories of the specific meadow fescue populations. In addition, although the present study focused on latitude, altitude is another major ecological factor which contributes to variation in flowering time (Orlandi et al. 2005). The relationship between altitude and nucleotide diversity still remains to be clarified.

Thirdly, as a large proportion of the polymorphisms identified in the present study were located in UTR and intron regions, it is possible that the polymorphic sites have been affected by indirect rather than direct selection pressures and that decay of LD over distances comparable to the length of a gene could decouple the causal and noncausal variants and lead to loss of diagnostic information. Although closely linked SNPs can be utilized for the purpose of crop breeding, identification of polymorphisms directly related to traits is essential for definitive understanding of plant biology and evolution. Further analysis is required for identification of such causal sequence polymorphisms.

Finally, geographical origin has been used in this study as a proxy for inferred phenotype. More detailed assessment of representative genotypes for heading date, vernalization requirement and cold tolerance would be required to test the various ecological hypotheses that have been proposed. Nonetheless, the comprehensive survey of gene-specific sequence variation presented here provides a solid foundation for such a study.

## Supporting Information

**Table S1.** Summary information for cleaved amplified polymorphic sequence (CAPS) genotyping of the meadow fescue genotypes. A and B stand for major and minor alleles identified in resequencing analysis, respectively.

**Table S2.** Allele frequency of the cleaved amplified polymorphic sequence (CAPS) marker in populations**.** A and B stand for major and minor alleles identified in the resequencing analysis, respectively.
